# Supplementary material for: The Anti-Inflammatory Effect of Low Molecular Weight Fucoidan from Sargassum siliquastrum in Lipopolysaccharide-Stimulated RAW 264.7 Macrophages via Inhibiting NF-κB/MAPK Signaling Pathways
Source: Mar Drugs. 2023 Jun 4;21(6):347. doi: 10.3390/md21060347 (PMC10303138; doi:10.3390/md21060347)
Supplement: Supplementary file 1 [file marinedrugs-21-00347-s001.zip › marinedrugs-2404655-supplementary.pdf]

Supplementary Material

Journal: Marinedrugs

Manuscript ID.: marinedrugs-2404655

Title of Paper: Anti-Inflammatory Effect of Low Molecular Weight Fucoidan from *Sargassum siliquastrum* in Lipopolysaccharide-Stimulated RAW 264.7 Macrophages via Inhibiting NF-κB/MAPK Signaling Pathways

Authors: Arachchige Maheshika Kumari Jayasinghe, Kirinde Gedara Isuru Sandanuwan Kirindage, Ilekuttige Priyan Shanura Fernando, Kil-Nam Kim, Jae-Young Oh, Ginnae Ahn *

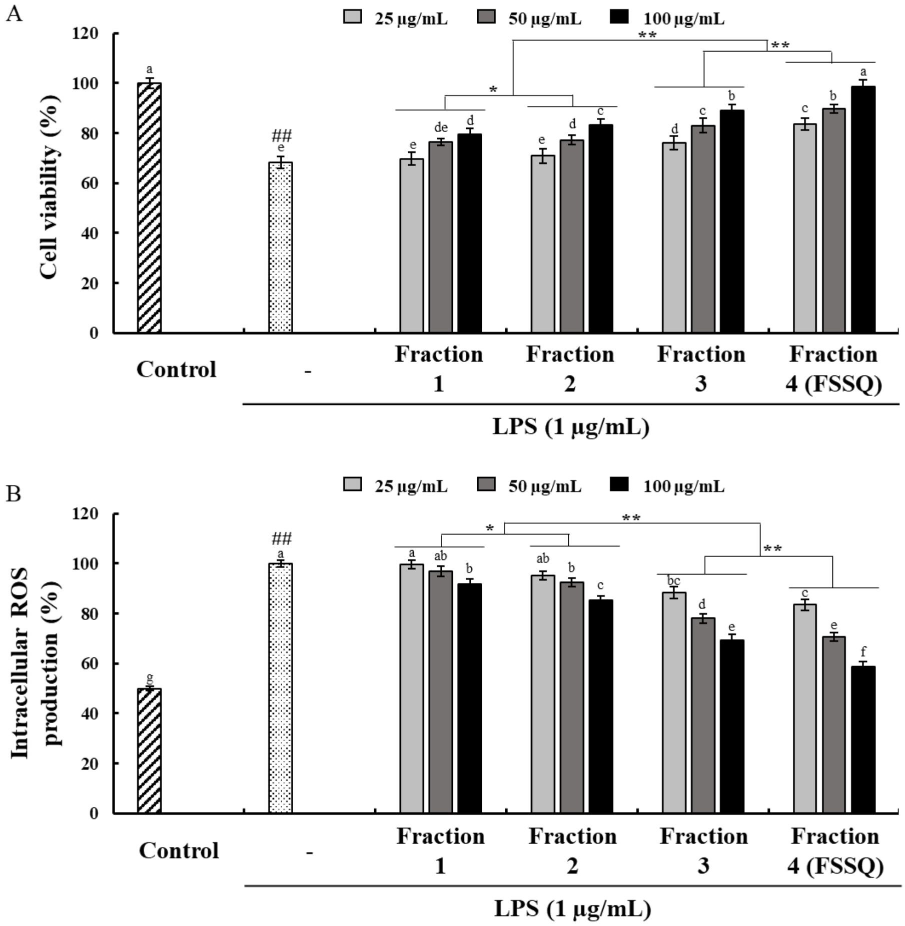

Figure S1. Effects of polysaccharide fractions on (A) cell viability and (B) intracellular ROS levels after LPS stimulation. Fraction 4 (FSSQ) indicated considerable cytoprotective effects in contrast to Fraction 1, 2, and 3. The values represent data from three distinct experiments (n=3) and are shown as the mean ± standard error (SE). Significantly different results were indicated with different lowercase English letters ($p < 0.05$). (*$p < 0.05$; **$p < 0.01$ vs the LPS-treated group, ## $p < 0.01$ in contrast to the control group).
